# Association of triglyceride-glucose index with sarcopenia: NHANES 2011–2014

**DOI:** 10.3389/fendo.2024.1452664

**Published:** 2024-09-23

**Authors:** Xue Wei, Dandan Liu

**Affiliations:** Department of Endocrinology, The Eighth Affiliated Hospital of Sun Yat-sen University, Shenzhen, Guangdong, China

**Keywords:** TyG index, sarcopenia, low muscle mass, low muscle strength, insulin resistance

## Abstract

**Background:**

A newly developed technique, the Triglyceride-glucose (TyG) index, supplies a more straightforward method to identify IR than the HOMA-IR (Homeostasis Model Assessment of Insulin Resistance). Yet no methodical analysis has looked into the link involving the TyG index and low muscle mass (LMM), low muscle strength (LMS), and sarcopenia within the US. Thus, this study intended to find any connection concerning the TyG index and LMM, LMS, and sarcopenia.

**Methods:**

Between 2011 to 2014, data from the NHANES were used to conduct a nationally representative study involving 2,504 participants. LMM, LMS, and sarcopenia were the outcome variables. Moreover, this positive correlation persists irrespective of age and gender.

**Results:**

The TyG index revealed a significant correlation with the prevalence of developing LMM (OR = 1.63(1.26–2.11), *p*=0.001), LMS (OR = 1.61(1.36–1.91), *p*<0.001) and sarcopenia (OR = 1.59 (1.23–2.07), *p*<0.001), after correcting for all variables. Utilizing smooth curve fitting alongside two-piecewise linear regression models, an inverted U-shaped correlation between the TyG index and the prevalence of LMM, LMS, and sarcopenia. Finally, subgroup analysis revealed that the association between the TyG index and LMM, LMS, and sarcopenia was particularly evident in all gender, age subgroups, and individuals with a normal BMI of 25.

**Conclusion:**

Sarcopenia and the TyG index reveal an essential positive link. It highlights the potential utility of the TyG index as a screening tool for identifying individuals at risk of sarcopenia earlier.

## 1 Introduction

Sarcopenia, involves the progressive and extensive decline of total muscle mass across the body, accompanied by reductions in muscle strength and physical function. CVD (cardiovascular disease), hypertension, T2DM, and obesity are more prevalent among individuals with sarcopenia (1–4).

The early stages of sarcopenia are usually asymptomatic, slow-progressing, and underappreciated its steadily increasing incidence. Studies have found that reduction in the incidence of sarcopenia could markedly decrease the financial impact on the US economy (5). According to a retrospective study of 4,021 adult Americans, individuals with sarcopenia have higher hospitalization rates and annual costs ($2,315.7 per person) compared to those without sarcopenia (6). Consequently, early identification of sarcopenia is essential for the success of treatments and for halting the advancement of the condition.

Aging, neuromuscular dysfunction, IR, and inflammation are all linked to the development of sarcopenia (7). Sarcopenia can be triggered by IR through the PI3K-AKT-mTORC pathway, as demonstrated by multiple research studies on animals (8–14). Insulin and amino acids play essential roles in promoting protein synthesis in skeletal muscle through the PI3K-AKT-mTORC pathway (8–12). Studies, such as the one conducted by Das et al., have demonstrated that IR can lead to stronger inhibition of mTORC1, resulting in reduced levels of protein synthesis in muscle (13). Additionally, research by Rong et al. using animal models has shown that IR can promote the production of inflammatory factors, leading to muscle atrophy through the PI3K-Akt pathway (14). A great deal cross-sectional investigations carried out across distinct countries suggest a noteworthy correlation between IR and sarcopenia (15–17). For instance, Park JH et al. reported a substantial inverse link between skeletal muscle mass and HOMA-IR (15). Similarly, a study involving Chinese cohorts have yielded similar results (16). However, it remains unclear whether the TyG index is associated with LGS. The method that is utilized most frequently to evaluate insulin sensitivity is the HOMA-IR. A newly developed technique, the TyG index, supplies a more straightforward method to identify IR than the HOMA-IR in diabetes, carotid atherosclerosis, arterial stiffness, and non-alcoholic liver disease (17–20). Yet no methodical analysis has looked into the link involving the TyG index and LMM, LMS and sarcopenia within the US.

Thus, the aim of this research attempted to seek out the connection between the incidence of LMM, LGS, sarcopenia and the TyG index.

## 2 Methods

### 2.1 Study population

The data were obtained from the NHANES. Biennially, a cohort of 5,000 residents gets picked from counties across the nation. Several sampling weights are allocated to each participant. Following a complex sample weighting process, these volunteers could represent the entire US population. The U.S. population from 2011 to 2014 was examined through a retrospective analysis of the NHANES. Each NHANES participant provided written informed consent.

**Figure 1:**
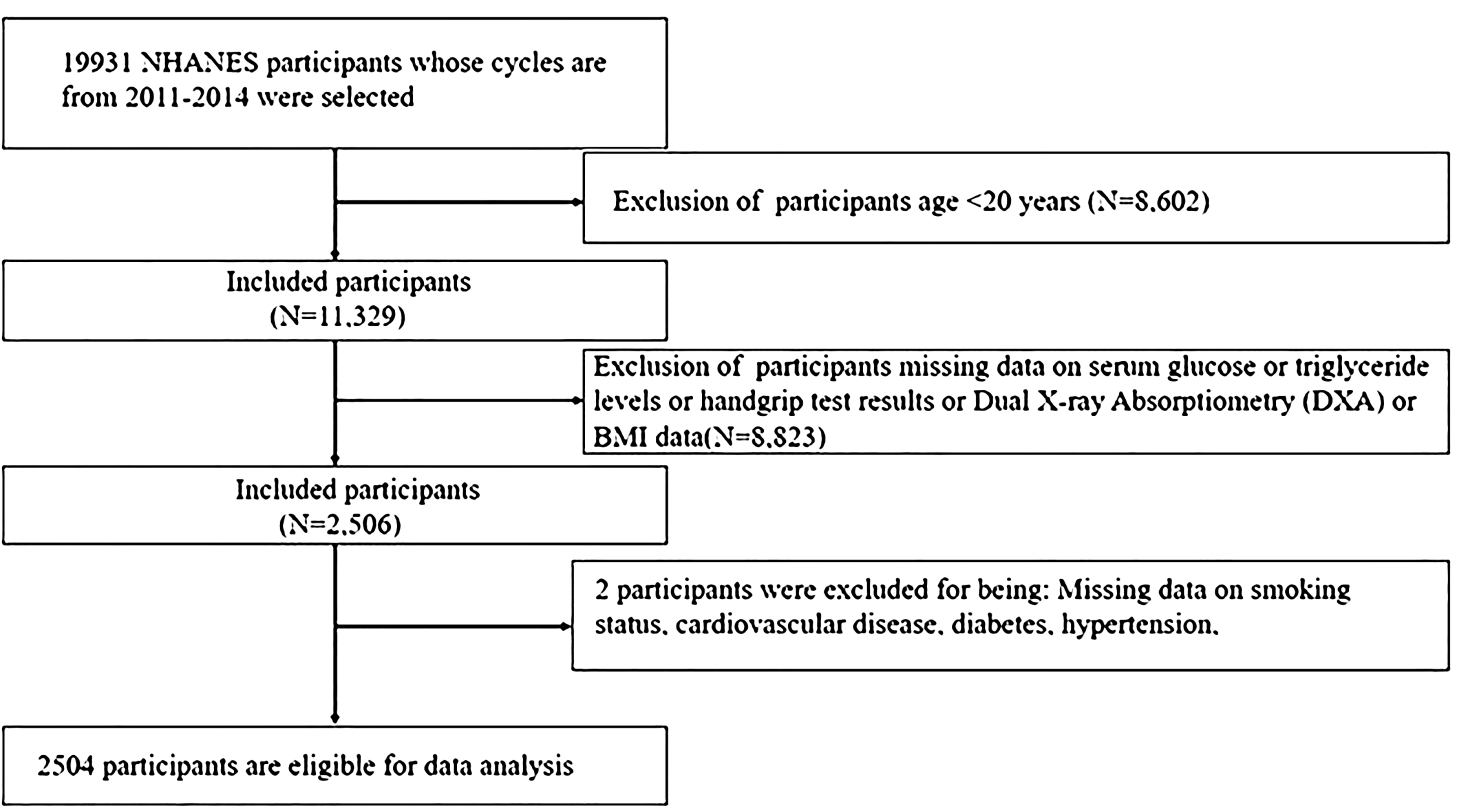
Flowchart of participants selection.

The study included 19,931 participants from 2011 to 2014 NHANES. A total of 17,427 individuals were disqualified for the reasons listed below: (1) Age <20 years (n=8,602); (2) Missing data on DXA (Dual X-ray Absorptiometry) or serum glucose or triglyceride levels or handgrip test results (HGS) (n=8,823); (3) Missing data on smoking status, CVD, T2DM, hypertension (n=2). Ultimately, the study encompassed 2,504 individuals (**Figure 1**).

### 2.2 Definition of the TyG index

The TyG index was calculated using the formula: TyG = ln [fasting triglycerides (mg/dL) × fasting plasma glucose (mg/dL)/2] (21). To facilitate a deeper examination, we handled the TyG index and subsequently segmented it into quartiles according to its values.

### 2.3 Muscle strength measurements

Muscle strength was evaluated using the Combined Grip Strength. The task required of participants was to stand with one hand tightly gripping a dynamometer. The test was conducted three times for each hand, with participants alternating hands between each test. A 60-second rest period was provided for the same hand between measurements. The Combined Grip Strength (kg) was calculated by summing the maximum grip strength values from each hand.

### 2.4 Definition of low muscle mass, low muscle strength, sarcopenia

ALM (appendicular lean mass), which is determined by totaling the lean tissues (excluding bone) from all four limbs. Appendicular skeletal muscle mass index (ASMI) was calculated by dividing ALM (kg) by weight (kg) (22, 23). Muscle strength, on the other hand, was expressed as adjusted hand grip strength (AHGS), which accounts for the influence of weight on grip strength. AHGS was calculated by dividing HGS (kg) by weight (kg) (24).

To define LMM, we used the criterion of an ASMI value that was one standard deviation below the sex-specific average for a reference cohort of adults in the NHANES dataset who were between the ages of 20 and 40. The cutoff points for LMM were 0.289 for men and 0.224 for women. The lowest quintiles of AHGS value, particular to a certain sex, were identified to be LMS. The cutoff points for low muscle strength were 0.897 for men and 0.637 for women. Individuals meeting the criteria for both LMM and LMS were classified as having sarcopenia.

### 2.5 Confounding variables

Age, gender, race, educational background, PIR (Poverty Income Ratio), and nicotine usage were self-reported by participants (25). By means of their weight and height, the BMI was derived. BMI was categorized into three distinct groups for this study: “Normal or low weight” for BMI values less than 25 kg/m^2, “Overweight” to a BMI of 25 to 29.9 kg/m^2, and “Obesity” to a BMI of 30 kg/m^2 or above. PIR was calculated by dividing family (or individual) income by poverty guideline. An official diagnosis of hypertension, the application of antihypertensive drugs, a DBP (diastolic blood pressure) of 90 mmHg or higher, and a SBP (systolic blood pressure) of 140 mmHg or higher are the markers of hypertension. Diabetes is characterized by a glycated hemoglobin (HbA1c) level of 6.5% or higher, being clinically diagnosed with diabetes, or currently using anti-diabetic medication. Clinically recognized disorders like chest pain, heart failure, and myocardial infarction are all included in CVD.

### 2.6 Statistical analysis

The study utilized percentages to represent categorical data and mean ± standard deviation (SD) to display continuous variables. Weighted chi-square tests and weighted linear regression models were to examine differences between categorical and continuous data, respectively. Based on the STROBE declaration (26), the link was evaluated using three distinct models. Model 1 failed to alter a single variable. Sex, age, ethnic background, education and PIR were elements taken for consideration in Model 2. Model 3 got into account variables such smoking status, diabetes, hypertension, height and CVD as well as to making changes from Model 2. Possible modifying effects of the TyG index involving sarcopenia were investigated by doing stratified multifactorial regression analysis on subgroups characterized by age, sex, and BMI. The assessment of heterogeneity among the subgroups was made possible by the inclusion of an interaction term. A linear trend test was utilized to verify the consistency of the correlations. Nonlinear interactions were evaluated using a Generalized Additive Model (GAM) with smooth curve fitting. An additional analysis was carried out utilizing the log-likelihood ratio to evaluate the model’s fit. Statistical analyses were conducted using R software (version 4.3.2) and Empower Stats. Statistical significance was set at *p* < 0.05.

## 3 Results

### 3.1 Participants’ characteristics

Enrollment in the study comprised 2,504 individuals, of whom the average age was 39.27 ± 0.41. Of these participants, 51.98% were men and 48.02% were women. The mean TyG index value was 8.53 ± 0.03. The results indicated a sarcopenia prevalence of 13.41%. Regarding age, educational background, ethnicity, smoking status and health conditions (which incorporates hypertension and T2DM), there were notable variations between the two groups (*p* < 0.05). Typically, individuals diagnosed with sarcopenia are older, smokers, higher educated, hypertension and diabetes. Additionally, a higher TyG index, lower ASMI, and lower AHGS were observed in individuals with sarcopenia compared to those without the condition. **Table 1** displays the included participants’ weighted demographic baseline information.

**Table 1:** Weighted baseline characteristics by Sarcopenia: NHANES 2011-2014.

	Non-Sarcopenia (2368)	Sarcopenia (136)	*P* value
**Age**	38.59 ± 0.44	43.64 ± 0.94	<0.001
**PIR**	2.86 ± 0.11	2.69 ± 0.16	0.225
**Height (m)**	1.70 ± 0.00	1.69 ± 0.01	0.326
**Sex (%)**			0.412
Male	52.43	49.06	
Female	47.57	50.94	
**Race (%)**			<0.001
Mexican American	9.36	11.71	
Other Hispanic	6.65	5.55	
Non-Hispanic white	63.15	72.04	
Non-Hispanic black	12.22	7.52	
Non-Hispanic Asian	6.02	1.16	
Other race	2.59	2.02	
**Education level (%)**			0.047
Less than 9th grade	2.99	5.14	
9th–11th grade	10.73	13.91	
High school graduate/GED or equivalent	19.68	23.53	
Some college or AA degree	33.49	32.53	
College graduate or above	33.11	24.89	
**Smoked ≥ 100 cigarettes in life (%)**			0.047
No	60.29	52.75	
Yes	39.71	47.25	
**CVD (%)**			0.269
No	96.98	95.19	
Yes	3.02	4.81	
**Diabetes (%)**			<0.001
No	94.45	74.74	
Yes	5.55	25.26	
**Hypertension (%)**			<0.001
No	65.43	44.56	
Yes	34.57	55.44	
**TyG index**	8.47 ± 0.02	8.90 ± 0.05	<0.001
**AHGS (kg/kg)**	1.01 ± 0.01	0.64 ± 0.01	<0.001
**ASMI (kg/kg)**	0.29 ± 0.00	0.24 ± 0.00	<0.001

Mean ± SD for continuous variables; proportion for categorical variables.

PIR, family income-to-poverty ratio; ASMI, appendicular muscle mass index; AHGS, adjusted hand grip strength; CVD, cardiovascular disease.

### 3.2 Association between the TyG index and the prevalence of LMM, LMS, and sarcopenia

The risk of developing LMM, LMS, and sarcopenia intensifies alongside an elevation in the TyG index is shown in **Table 2**. In the first crude model, the TyG index indicated a strong correlation with the chance to generate LMM (OR = 2.03 (1.67–2.47), *p* < 0.001), LMS (OR = 2.25 (1.98–2.56), *p* < 0.001) and sarcopenia (OR = 2.30(1.90–2.78), *p* < 0.001). Interestingly, out of all the groups, the sarcopenia group had the greatest OR. When all confounders were taken into account, these results didn’t change. For the purposes of sensitivity research, the TyG index was then split into multiple categories. In fully adjusted model, the highest quartile of the TyG index indicates a stronger significant correlation with the prevalence of LMM (OR = 3.05(1.86–5.01), *p*<0.001), LMS (OR = 2.66(1.73–4.08), *p*<0.001) and sarcopenia (OR = 4.14 (1.92–8.14), *p*<0.001) compared to the lowest quartile.

**Table 2:** Logistic regression analysis results for the associations between TyG index and LMM, LMS, and sarcopenia (weighted).

	Model 1	Model 2	Model 3
Sarcopenia (OR (95%CI), *P* value)			
Continuous variables			
TyG	2.30(1.90–2.78) <0.001	2.15 (1.70–2.71) <0.001	1.59 (1.23–2.07) 0.002
Categorical variable			
Q1	Ref	Ref	Ref
Q2	2.29 (1.20–4.38) 0.018	2.03 (1.11–3.72) 0.035	1.93 (0.96–3.86) 0.060
Q3	3.62(1.86–7.05) <0.001	3.06 (1.68–5.59) 0.002	2.81 (1.38–5.71) 0.007
Q4	7.26 (3.95–13.35) <0.001	6.27 (3.52–11.17) <0.001	4.14 (1.92–8.14) <0.001
**P for trend**	<0.001	<0.001	0.002
Low muscle mass (OR (95%CI), *P* value)			
Continuous variables			
TyG	2.03 (1.67–2.47) <0.001	1.90(1.49–2.41) <0.001	1.63(1.26–2.11) 0.001
**Categorical variable **			
Q1	Ref	Ref	Ref
Q2	1.98(1.36–2.87) <0.001	1.73(1.18–2.54) 0.012	1.71(1.09–2.69) 0.022
Q3	2.82(1.89–4.21) 0.001	2.34(1.55–3.54) <0.001	2.21(1.40–3.51) 0.003
Q4	4.48(3.13–6.42) <0.001	2.34(2.51–5.99) <0.001	3.05(1.86–5.01) <0.001
P for trend	<0.001	<0.001	0.001
Low muscle strength (OR (95%CI), *P* value)			
Continuous variables			
TyG	2.25 (1.98–2.56) <0.001	2.11(1.80–2.46) <0.001	1.61(1.36–1.91) <0.001
**Categorical variable **			
Q1	Ref	Ref	Ref
Q2	1.70(1.06–2.72) 0.037	1.54(0.97–2.46) 0.085	1.43(0.89–2.31) 0.166
Q3	2.60(1.75–3.88) <0.001	2.32(1.60–3.36) <0.001	2.13(1.43–3.17) 0.003
Q4	4.69(3.15–6.96) <0.001	4.03(2.72–5.96) <0.001	2.66(1.73–4.08) <0.001
P for trend	<0.001	<0.001	<0.001

Model 1: No adjusted.

Model 2: Adjusted by age, gender, race, education level, PIR.

Model 3: Adjusted by age, gender, race, education level, smoking status, PIR, height, diabetes, hypertension, and CVD.

### 3.3 Nonlinear associations between the TyG index and the prevalence of LMM, LMS, and sarcopenia

**Figure 2:**
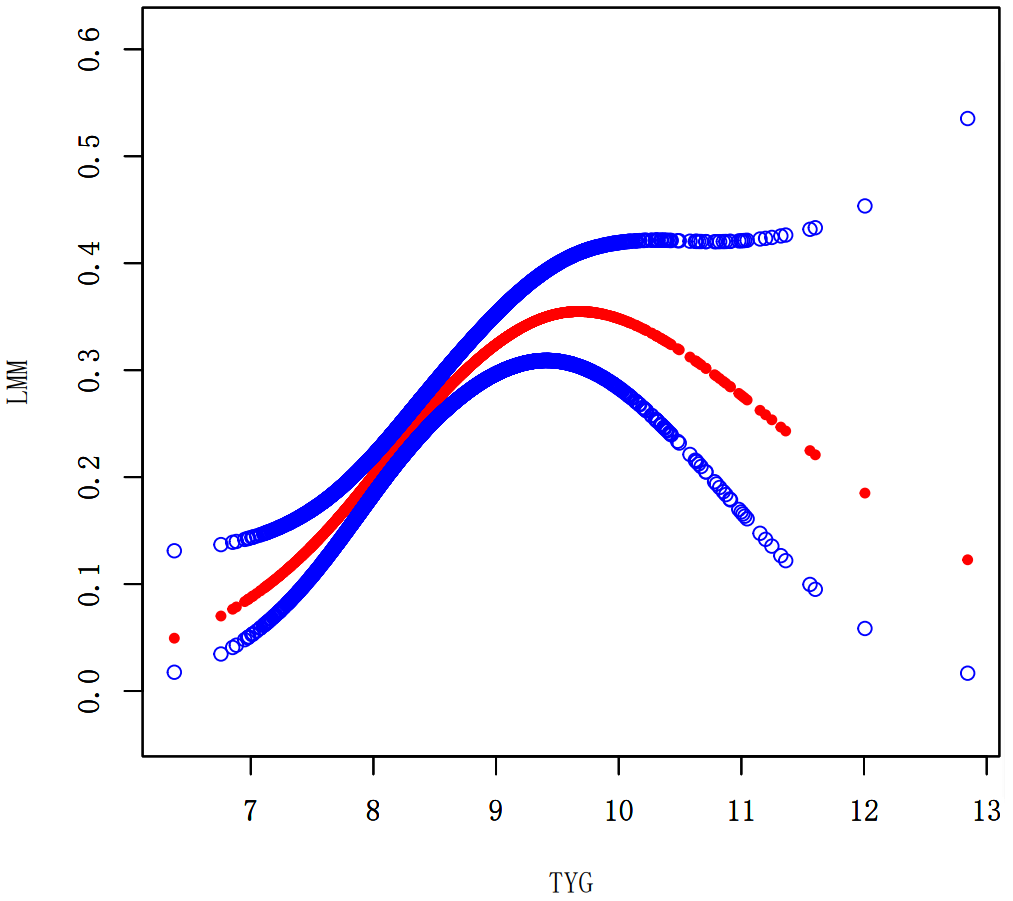
Smooth curve fitting using GAM to evaluate the nonlinear relationship between the TyG index and the risk of LMM.

**Figure 3:**
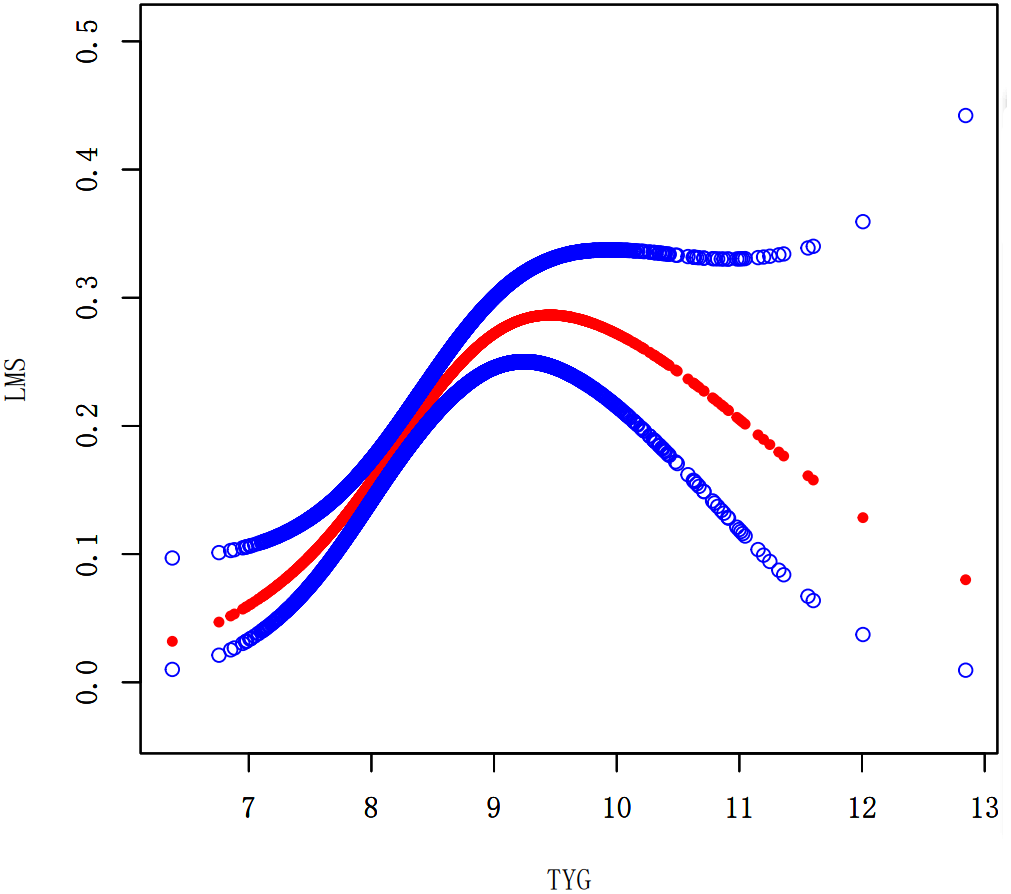
Smooth curve fitting using GAM to evaluate the nonlinear relationship between the TyG index and the risk of LMS.

**Figure 4:**
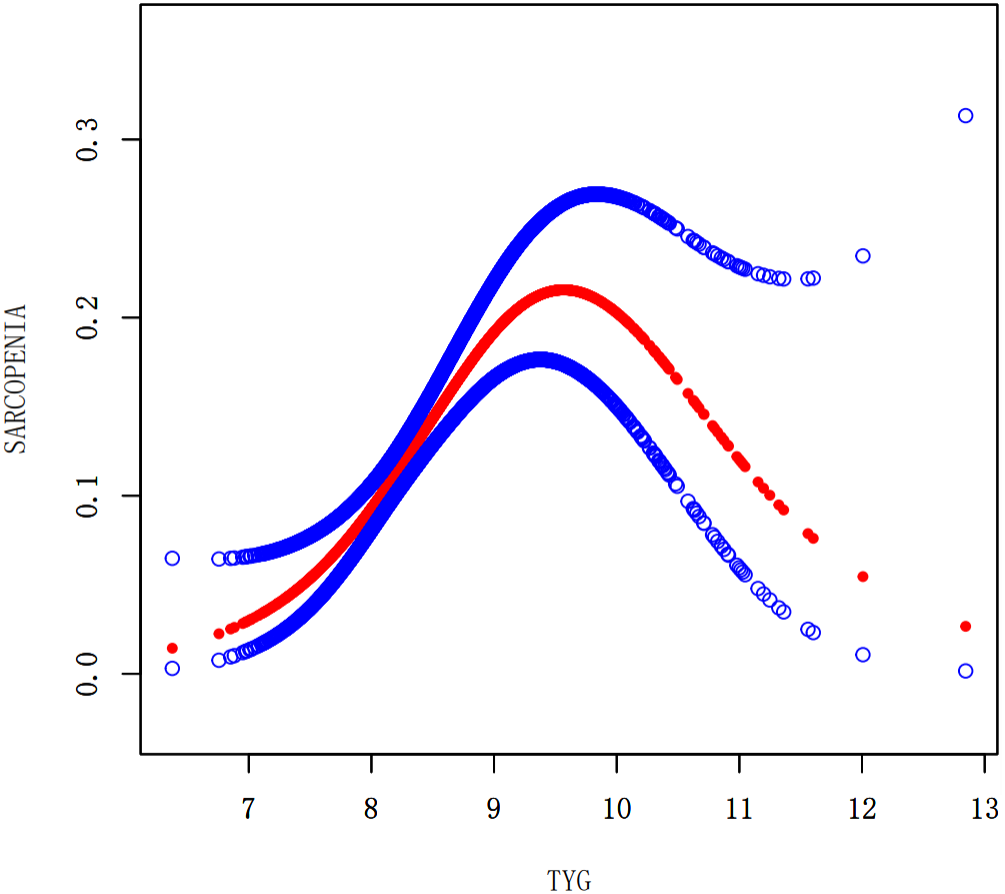
Smooth curve fitting using GAM to evaluate the nonlinear relationship between the TyG index and the risk of Sarcopenia.

We performed a smooth curve fitting to describe the nonlinear connection between the TyG index and the prevalence of LMM, LMS, and sarcopenia (**Figures 2**–**4**). Using a two-segment linear regression model, the prevalence of LMM, LMS, and sarcopenia was discovered to be an inverted U-shaped relationship with the TyG index (**Table 3**). The risk of LMM, LMS, and sarcopenia boosted rapidly with the increase in the TyG index before reaching the turning point (Tyg index= 8.15 for LMM, TyG index = 8.98 for LMS, TyG index = 9.16 for sarcopenia). Beyond these turning points, the risk of these conditions with further increases in the TyG index appeared to dampen. We believe that the smoothing component observed after the inflection point in the relationship between the TyG index and the prevalence of LMM, LMS, and sarcopenia may be attributed to missing data in patients with a high TyG index. It is possible that individuals with a higher TyG index, who may be more susceptible to sarcopenia, were less likely to participate or complete the assessments, leading to missing data.

**Table 3:** Threshold effect of evaluation indicators of the TyG index on LMM, LMS, and sarcopenia using a two-piecewise linear regression model.

	OR (95% CI)	*P* value
Sarcopenia		
Fitting by two-piecewise linear model		
**Breakpoint**	9.16	
< 9.16	2.73(1.99–3.74)	<0.001
> 9.16	0.63 (0.41–0.96)	0.034
**Log-likelihood ratio**	<0.001	
Low muscle mass		
Fitting by two-piecewise linear model		
**Breakpoint**	8.15	
< 8.15	2.01(1.64–2.47)	<0.001
> 8.15	0.54 (0.64–0.90)	0.017
**Log-likelihood ratio**	<0.001	
Low muscle strength		
Fitting by two-piecewise linear model		
**Breakpoint**	8.98	
< 8.98	2.34 (1.82–3.02)	<0.001
> 8.98	0.67 (0.46–0.96)	0.031
Log-likelihood ratio	<0.001	

All models were adjusted for age, gender, race, education level, smoking status, PIR, height, diabetes, hypertension, and CVD.

### 3.4 Subgroup analysis

To identify any potential variations within particular subpopulations and to verify the coherence underlying the link involving the TyG index the prevalence of LMM, LMS, and sarcopenia, subgroup analyses and interaction tests were executed (**Table 4**). These analyses involved stratifying the data by age, gender, and BMI. Interaction analyses indicated that the connection between the TyG index and sarcopenia was influenced by BMI and age rather than gender. In contrast, for participants with LMM or LMS, neither age nor gender appeared to affect this association. The TyG index was positively correlated with LMM, LMS, and sarcopenia prevalence across all gender, age subgroups, and individuals with a normal BMI of 25. Interestingly, this association was not observed in participants who had a BMI of 25 or higher.

**Table 4:** Stratified associations between TyG index and sarcopenia and low muscle strength (weighted).

Stratification	OR (95% CI)	*P* value	*P* for interaction
Sarcopenia			
**Gender**			0.709
Male	1.54(1.09, 2.17)	0.030	
Female	1.661.28, 2.15)	0.002	
**Age**			0.005
20-40	2.21(1.60, 3.07)	<0.001	
≥40	1.33(1.04, 1.72)	0.043	
**BMI**			0.016
<25	3.14(1.43, 6.89)	0.017	
25-30	1.15(0.76, 1.72)	0.526	
≥30	1.01(0.73,1.39)	0.969	
Low muscle mass			
**Gender**			0.692
Male	1.57(1.14, 2.17)	0.017	
Female	1.71(1.27, 2.31)	0.004	
**Age**			0.089
20-40	1.98(1.55, 2.54)	<0.001	
≥40	1.45(1.07, 1.98)	0.033	
**BMI**			0.016
<25	2.39(1.25, 4.59)	0.025	
25-30	0.89(0.65, 1.21)	0.475	
≥30	1.09(0.77,1.56)	0.633	
Low muscle strength			
**Gender**			0.191
Male	1.47(1.17, 1.85)	0.006	
Female	1.86(1.43, 2.42)	<0.001	
**Age**			0.139
20-40	1.92(1.46, 2.51)	<0.001	
≥40	1.45(1.16, 1.82)	0.007	
**BMI**			0.015
<25	3.72(1.37, 10.05)	0.027	
25-30	1.16(0.92, 1.45)	0.239	
≥30	0.96(0.71,1.30)	0.783	

All models were adjusted for age, gender, race, education level, smoking status, PIR, height, diabetes, hypertension, and CVD.

## 4 Discussion

To An innovative technique to IR is the TyG index. The bond involving the TyG index and conditions, including diabetes, hypertension, and metabolic syndrome, has already been evaluated in research that has been published. However, research on the TyG index’s relationship with LMM, LMS and sarcopenia is limited. The TyG index were positively and independently associated with LMM and LMS in this research. Notably, when both LMM and LMS were present, indicating a sarcopenic state, the OR was even higher. Importantly, these associations remained robust despite adjusting all variables that caused confusion. Furthermore, the research found an inverted U-shaped association between the TyG index and the prevalence of LMM, LMS, and sarcopenia. The results suggested that the potential utility of the TyG index as a screening tool for identifying individuals at risk of sarcopenia earlier.

The causal relationship between IR and sarcopenia is not yet fully understood. However, multiple studies have indicated that IR and muscle loss can interact and reinforce each other. Sandra et al. demonstrated that IR impairs the translocation of the glucose transporter (GLUT)-4, hindering glucose absorption in skeletal muscle (9, 27). This impairment leads to increased protein hydrolysis and decreased protein synthesis, potentially reducing muscle mass and strength (28). Furthermore, María et al. demonstrated that fat accumulation in skeletal muscle leads to lipotoxicity and the secretion of proinflammatory myokines, resulting in IR and skeletal muscle atrophy (29). Decreased muscle mass disrupts glucose storage, further impairing insulin sensitivity. Thus, IR is intricately associated with sarcopenia.

Sarcopenia is a syndrome characterized by reduced muscle mass and strength. It has been suggested that focusing solely on low muscle mass may be too narrow and potentially of limited clinical value (30, 31). Our study findings align with this perspective, as we observed that the TyG index exhibited higher OR for sarcopenia compared to LMM or LMS. Therefore, regular monitoring of the TyG index might thus be of equal importance in the prevention of LMM and LMS.

The correlation between sarcopenia and the TyG index is debatable. Park JH et al. reported a substantial inverse link between skeletal muscle mass and IR while examining information collected from 372,399 Korean volunteers (15). While, an opposite correlation was observed between the TyG index and sarcopenia in the Chen et al. study, which involved 1819 Chinese adults (16). Variability in a sample’s proportion, race, methods of investigation, and the disparities in databases used in multiple investigations may account for this discrepancy. Furthermore, the variation in age ranges across three studies may restrict the universality of the outcomes.

We performed a stratified analysis by sex, age, and BMI in our study. Interestingly, we observed that there was no association between the TyG index and sarcopenia in the obesity group. The results of our study are inconsistent with findings from a study conducted in South Korea, which demonstrated an association between obesity-related IR and the development of sarcopenia (17). The discrepancy in findings between our study and the South Korean study may be attributed to several factors, including differences in the age and race/ethnicity of the study populations. The South Korean study focused on individuals aged 60 years and older, specifically Korean individuals, while our study included individuals aged 20-59 years from diverse ethnic backgrounds. Additionally, obesity boosts the likelihood of oxidative stress and ongoing inflammation, both of which may have a variety of effects on TyG index levels (32–34). Notably, our study revealed gender disparities, with females having a higher prevalence of LMM, LMS, and sarcopenia compared to males. This finding aligns with a prior study conducted in Korean populations, which also reported a higher risk of these conditions in females (17). These gender differences may be influenced by sex hormones. Postmenopausal women, for example, often experience a rapid increase in visceral fat and a decrease in insulin sensitivity, leading to insulin resistance (35). Alterations in estrogen levels before and after menopause may partially contribute to the significant association observed between the TyG index and incident sarcopenia in females.

There are some abundances of drawbacks with this investigation which deserve to be emphasized. Initially, this investigation’s cross-sectional methodology means that it fails to show causality involving sarcopenia and the TyG index. Prospective cohort researches are required to determine causality. Additionally, the study was limited to those who had fasting triglyceride and glucose measures available and had undergone DXA testing raises the possibility of selection bias as well. This bias is further exacerbated by the exclusion of some possible factors that were not taken into account for the modifications. It is inevitable that this circumstance will lead to bias. Thirdly, racial differences may influence the results, given the reliance on the NHANES database for this study. Lastly, the study did not account for other potential confounding factors, which may also serve as sources of bias.

## 5 Conclusion

Our study has demonstrated that the risk of developing sarcopenia intensifies alongside an elevation in the TyG index among diverse age categories and genders. These findings highlight the potential utility of the TyG index as a screening tool for identifying individuals at risk of sarcopenia earlier. Early detection can lead to timely initiation of treatment, which may help improve outcomes and quality of life for individuals affected by sarcopenia and potentially reduce social and healthcare expenditures associated with its management.

## Data Availability

The original contributions presented in the study are included in the article/supplementary material. Further inquiries can be directed to the corresponding author.
